# Current status of proton pump inhibitor usage in patients with acute coronary syndrome and atrial fibrillation: a cross-sectional study

**DOI:** 10.3389/fcvm.2026.1601616

**Published:** 2026-04-15

**Authors:** Ying Bai, Jianqi Wang, Guangyao Li, Zhen Zhou

**Affiliations:** 1 Department of Pharmacy, Beijing Tongren Hospital, Capital Medical University, Beijing, China; 2 Department of Cardiovascular Center, Beijing Tongren Hospital, Capital Medical University, Beijing, China; 3 School of Biomedical Engineering, Capital Medical University, Beijing, China

**Keywords:** acute coronary syndrome, antithrombotic treatment, atrial fibrillation, oral anticoagulant, proton pump inhibitor

## Abstract

**Background:**

The real-world status of proton pump inhibitor (PPI) administration in patients with acute coronary syndrome (ACS) and atrial fibrillation (AF) remains largely unknown.

**Objectives:**

This study aimed to analyze the prescription pattern, trends and influencing factors of PPIs among Chinese patients with ACS and AF at discharge.

**Methods:**

This single-center, retrospective, cross-sectional study included patients with ACS and AF who were hospitalized at the Cardiovascular Center of Beijing Tongren Hospital from January 2010 to December 2018. All patients were subsequently categorized into two groups based on PPI administration at discharge (i.e., the PPI and non-PPI group), after which factors influencing PPI use were analyzed.

**Results:**

This study included 531 patients diagnosed with ACS and AF, with a median age of 73 years, 61.4% of these patients were male. Among them, 254 patients (47.8%) were administered PPIs. A significant increasing trend in PPI administration was observed from 21.3% (2010–2012) to 63.5% (2016–2018) (*p* < 0.001). Patients receiving PPIs were more likely to have non-ST-segment elevation myocardial infarction (UNSTEMI) [adjusted odds ratio (OR) 3.358, 95% confidence interval (CI) 1.819–6.196, *p* < 0.001], ST-elevation myocardial infarction (STEMI) [adjusted OR 4.092, 95% CI 2.177–7.694, *p* < 0.001], paroxysmal AF [adjusted OR 1.732, 95% CI 1.022–2.936, *p* = 0.041], GI disorders [adjusted OR 17.625, 95% CI 9.783–31.751, *p* < 0.001], were more likely to undergo catheter ablation [adjusted OR 13.368, 95% CI 3.836–46.586, *p* < 0.001] and more likely to receive oral anticoagulants [adjusted OR 1.918, 95% CI 1.006–3.658, *p* = 0.048] compared to those not receiving PPIs.

**Conclusions:**

Our findings revealed that approximately 50% of patients with AF and ACS were prescribed PPI at discharge, highlighting the need to carefully evaluate the risks and benefits of combining antithrombotic medications and PPIs on an individual basis.

## 1 Introduction

Coronary heart disease (CHD) and atrial fibrillation (AF) share many common risk factors, such as smoking, obesity, hypertension, and diabetes, which frequently coexist. Acute coronary syndrome (ACS) is an acute manifestation of CHD characterized by ST-elevation myocardial infarction (STEMI), non-STEMI (NSTEMI), and unstable angina (UA). Approximately 20% of patients with AF develop ACS or undergo percutaneous coronary intervention (PCI) (1). Current guidelines recommend dual antiplatelet therapy (DAPT) with aspirin and P2Y12 inhibitors as the standard treatment for ACS (2, 3). Although AF increases the risk of stroke and systemic embolism, anticoagulation therapy has been proven beneficial for reducing this risk (4–6). Guidelines recommend anticoagulation for patients with AF who are at high risk of stroke (7). Moreover, evidence has shown that the combined use of antiplatelet and anticoagulant agents reduced stroke and cardiovascular events in patients with ACS and AF but was associated with an increased risk of bleeding (8). Triple therapy (DAPT plus anticoagulation) had been identified one of the independent predictors of gastrointestinal bleeding (GIB) (9). One study revealed that antithrombotic agents accounted for the largest proportion of emergency hospitalizations for adverse drug events in the United States, with bleeding being the leading cause of hospitalization (10). Hospitalizations for GIB have increased in prevalence with the growing aging population receiving antithrombotic agents (11). Hence, prevention remains the best approach toward reducing the risk of GIB.

Proton pump inhibitors (PPIs) are the most potent acid-suppressing drugs predominantly prescribed for gastroesophageal reflux disease, gastroduodenal ulcers, GIB, etc. Existing evidence indicates that PPIs significantly reduced the incidence of GIB in patients with ACS who are receiving DAPT (12). Although current guidelines recommend the combined use of PPIs for patients receiving DAPT or triple antithrombotic therapy, their use in actual clinical practice has been lacking (13–15). This inadequacy in use may be attributed to the numerous adverse events associated with long-term PPI treatment despite its superior efficacy in reducing gastric acid compared with H2 receptor antagonists (H2RAs). A cohort study indicated that the risk of incident hip fracture among individuals using PPIs was higher than that in those using H2RAs. This increased risk may be attributed to the fact that PPIs are generally more effective than H2RAs in inhibiting an acidic gastric environment, which could promote greater detrimental effects on calcium and vitamin B12 absorption, as well as increased hypergastrinemia (16). Furthermore, a meta-analysis revealed a 1.26-fold increase in the risk for a more than 30% decline in glomerular filtration rate (GFR) with PPI use compared to H2RAs. Notably, an increase in cumulative length of exposure to PPIs was associated with a higher risk for PPI-related chronic kidney disease (CKD) and end-stage renal disease (17). Additionally, a case-control study found that individuals prescribed PPIs, but not an H2RA, exhibited increased risk for pneumonia (18). The use of PPIs has also been linked to increased mortality and hepatic decompensation among patients with liver cirrhosis, as well as an elevated risk for hepatic encephalopathy (19, 20). Moreover, a previous study identified a positive association between PPI use and various conditions, including stroke, falls, Clostridium difficile infections, asthma, pancreatic cancer, Parkinson's disease, anemia, and depressive disorders (21, 22). Furthermore, although PPIs significantly reduce the risk of bleeding, their potential impact on cardiovascular outcomes remains a matter of controversy (23, 24).

In patients with ACS who also have comorbid AF, inadequate use of PPIs can elevate the risk of GIB, whereas excessive PPI usage may lead to a range of adverse reactions. Both insufficient and excessive PPI use can pose an increased economic burden on patients and healthcare systems. The previous focus on PPI utilization within this specific patient population has been relatively limited, and there are currently vague evaluation criteria for PPI administration in these individuals. For example, the selection and duration of PPIs across different antithrombotic treatment regimens or varying comorbid conditions remain unclear, necessitating further in-depth exploration.

The real-world status of PPI administration among Chinese patients with ACS and AF remains largely unknown. Therefore, the current study aimed to identify the actual status of PPI administration among patients with ACS and AF from 2010 to 2018 and identify the factors influencing PPI administration at discharge. Understanding the current situation is the first step toward promoting the rational use of PPIs. We hope that this will enhance the understanding of PPI usage and inspire additional research aimed at improving the current clinical situation.

## 2 Material and methods

### 2.1 Study design

This retrospective, single-center, cross-sectional study included adults with ACS and AF who were admitted to the Cardiovascular Center of Beijing Tongren Hospital, a 1,700-bed tertiary care teaching hospital in China, from January 2010 to December 2018. The cardiovascular center ward had >100 beds. Our study protocol was approved by the Ethics Committee of Beijing Tongren Hospital. The need for informed consent was waived due to our use of retrospective medical records (TRECKY2019-124).

### 2.2 Study population

This study enrolled 701 patients diagnosed with ACS complicated with AF. ACS was defined to include UA, STEMI, NSTEMI as hospital discharge diagnoses. AF was also defined based on discharge diagnosis. After excluding cases with AF (mechanical heart valves or moderate to severe mitral stenosis) (*n* = 13), cases with incomplete data (i.e., those lacking discharge records or auxiliary examinations, *n* = 3), deaths during hospitalization (*n* = 52), no medication at discharge (*n* = 13), and duplicated cases (*n* = 89), 531 patients were ultimately included in the analysis of factors influencing PPI administration during hospitalization. For patients admitted multiple times, only the first hospitalization was considered. The case ID was utilized as the key filter to remove duplicate records. The included participants were then categorized into two groups based on PPI administration at discharge.

### 2.3 Data sources

Demographic and clinical data, including age, gender, past medical history, admission systolic blood pressure, diagnoses, and treatment strategies at discharge, were collected from the electronic information system. Additionally, the relevant laboratory data at admission, including hemoglobin, platelet count, serum creatinine, and liver function indicators, were documented. GI disorders, including digestive tract ulcers, gastritis, enteritis, Helicobacter pylori infection, gastroesophageal reflux, and GIB were recorded. The eGFR was calculated using the CKD-Epidemiology Collaboration (CKD-EPI) formula (25); stroke risk was estimated using the CHA_2_DS_2_-VASc scores; and bleeding risk was estimated using HAS-BLED scores (7). Trained researchers gathered all information using standard collection forms. Two researchers independently collected data and cross-checked each other's forms for errors, with another researcher subsequently verifying the collected data.

### 2.4 Statistical analyses

Normally distributed continuous variables were expressed as means ± standard deviations, whereas non-normally distributed variables were presented as medians ± interquartile ranges. Normal distribution of continuous variables was assessed by the Kolmogorov-Sirnov test. Categorical variables were presented shown as percentages (frequency). The *χ*2 test or Fisher's exact test was used to compare categorical variables among different groups. To identify the risk factors associated with the use of PPI, a binary logistic regression analysis was conducted. Variables that demonstrated statistical significance (*p* < 0.05) in the univariable analysis were subsequently incorporated into the multivariable regression model. The selection of risk factors in the present study was based on their clinical significance and the findings of previous studies. Data analysis was performed using the Statistical Package for Social Sciences version 22.0, with a *p* value of <0.05 indicating statistical significance.

## 3 Results

### 3.1 PPI use in patients with ACS and AF

**Figure 1:**
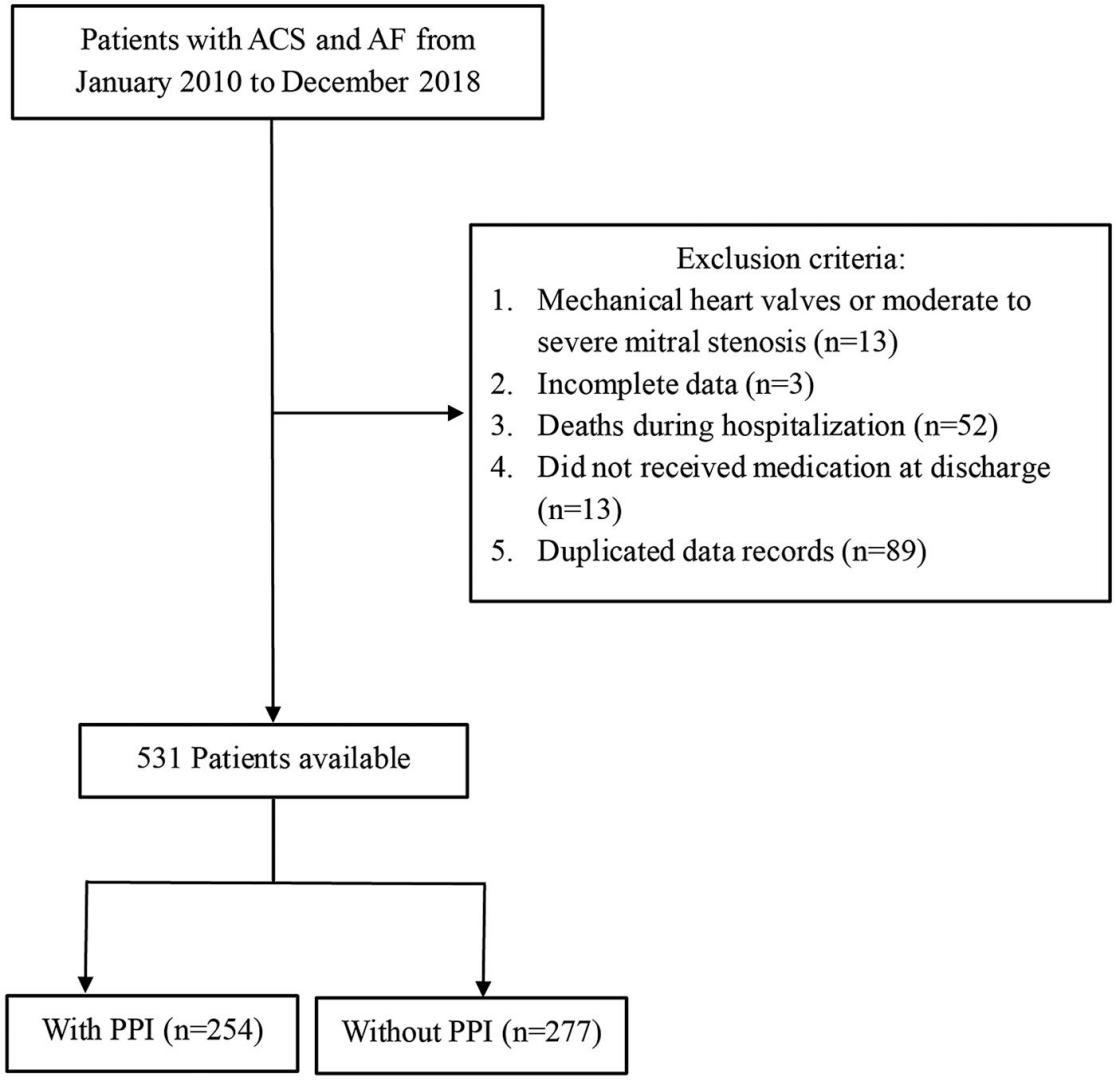
Study population flow chart.

This study included 531 patients with ACS and AF (median age, 73 years; 61.4% males), 254 (47.8%) received PPIs (Figure 1). Patients aged >65 years accounted for 70.6%. Our findings revealed that UA and paroxysmal AF were the most prevalent types of ACS and AF, respectively. Hyperlipidemia, hypertension, and diabetes mellitus were the three most predominant comorbidities. The most commonly used PPI was pantoprazole (87.8%), followed by esomeprazole (8.7%), rabeprazole (2.0%), and omeprazole (1.6%).

**Figure 2:**
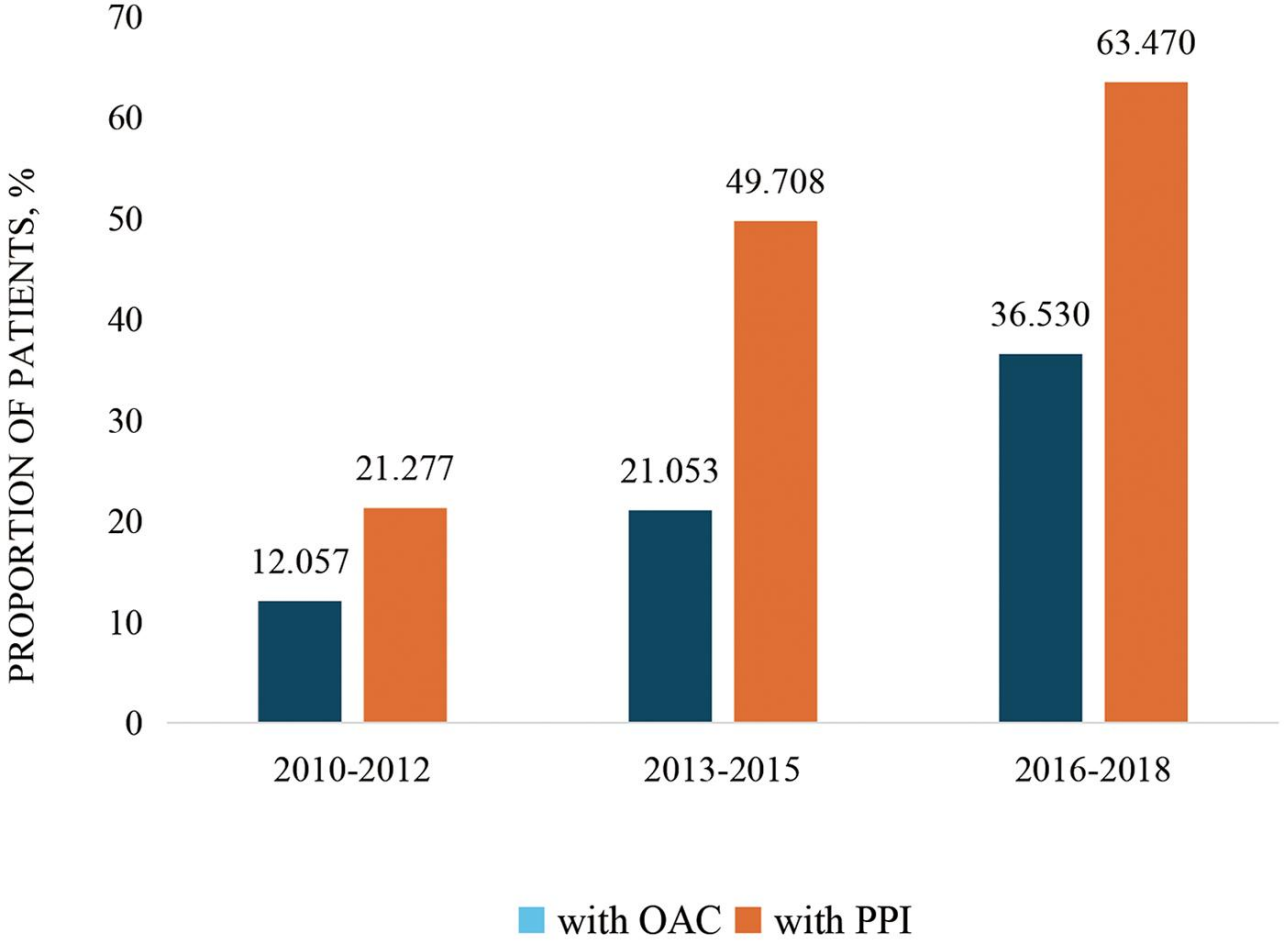
OAC and PPI usage trends in Chinese patients with ACS and AF.

**Figure 3:**
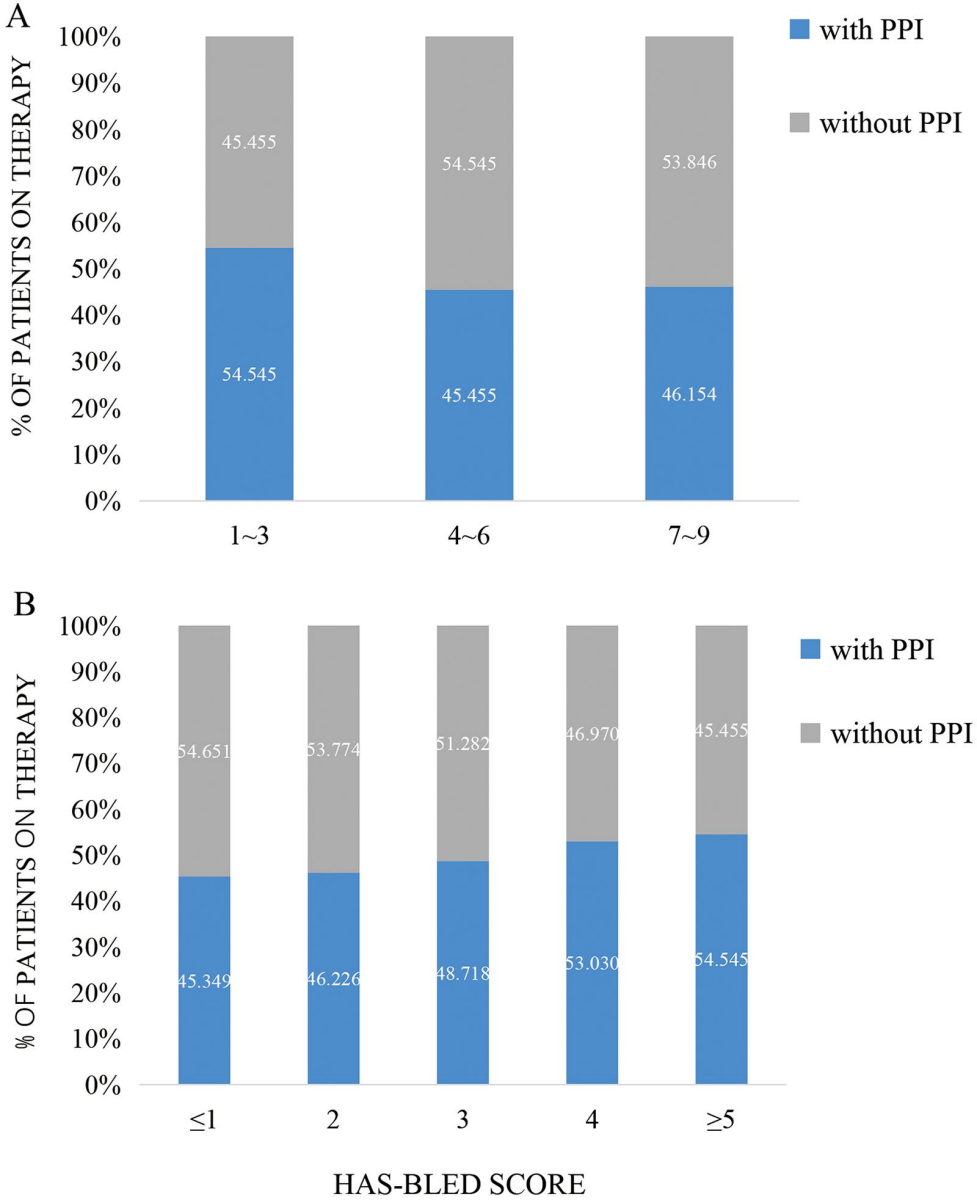
Use of PPIs and risk of CHA2DS2-VASc score **(A)** and HAS-BLED **(B)**.

The population was categorized into three groups (2010–2012, 2013–2015, and 2016–2018) according to the year of admission. An increasing trend was observed in the administration of oral anticoagulants (OAC) (from 12.1% to 36.5%; *p* < 0.001) and PPIs (from 21.3% to 63.5%; *p* < 0.001) (Figure 2). Our findings revealed that 96.2% of the patients were at high risk for stroke (CHA_2_DS_2_-VAS_C_ score of ≥2) and that 43.9% of patients were at high risk for bleeding (HAS-BLED score of ≥3) (Figure 3).

**Figure 4:**
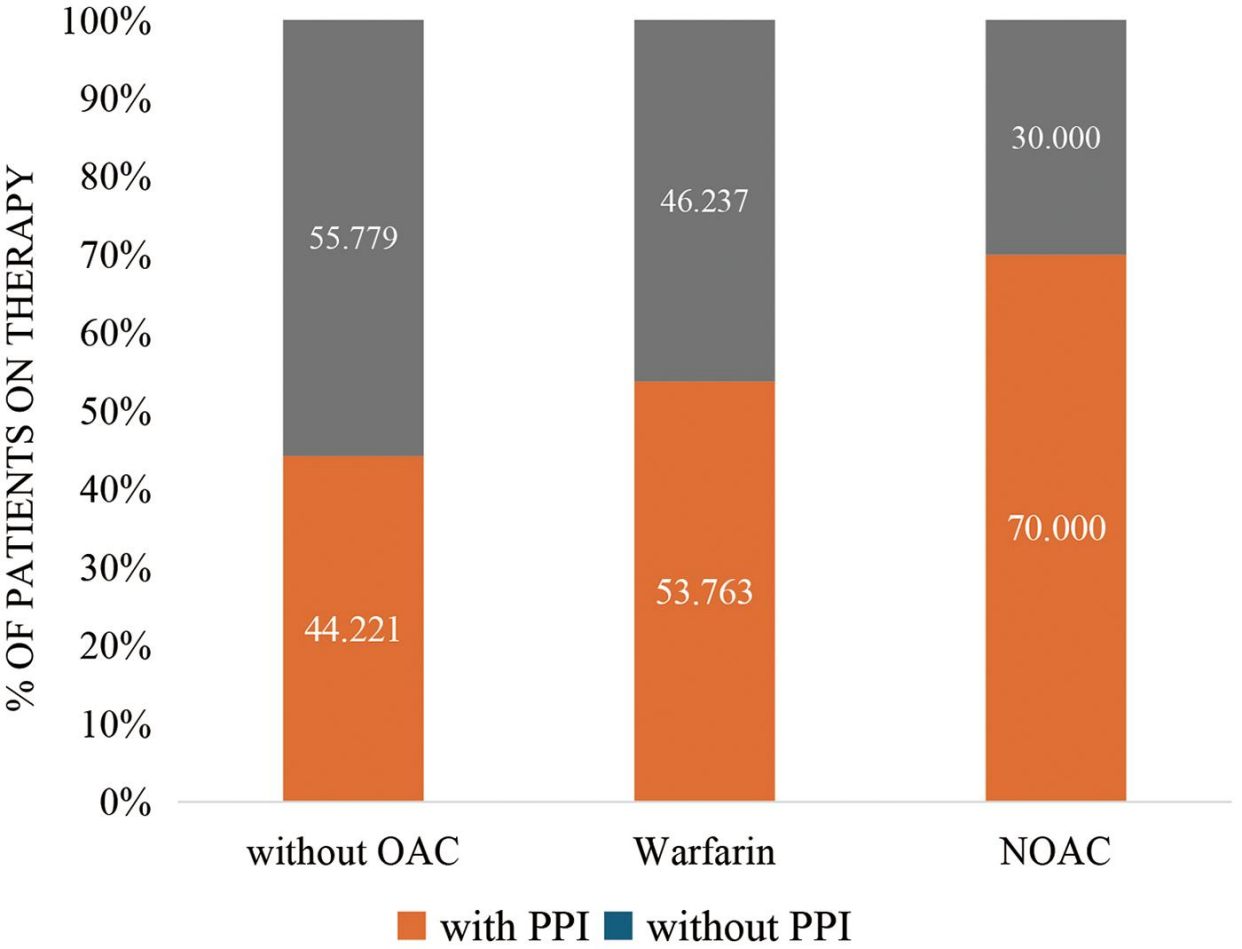
Use of PPIs and OAC therapy.

Regarding the use of antithrombotic agents, 4 (0.8%), 64 (12.1%), 330 (62.1%), 38 (7.2%), 55 (10.4%), and 40 (7.5%) patients received no antithrombotic agents, single antiplatelet treatment (SAPT), DAPT, OAC, OAC + SAPT, and OAC + DAPT, respectively. Among the 133 (25.0%) patients who received OAC, 93 and 40 received warfarin and NOAC, respectively. No difference in the incidence of GI was found between the OAC and non-OAC groups (36.8% vs. 31.2%; *p* = 0.226), although the OAC group was prescribed more PPIs (58.6% vs. 44.2%; *p* < 0.004). The proportion of PPI prescription was highest in the NOAC groups, followed by the warfarin group and non-OAC group (70.0%, 53.8%, and 44.2%; *p* = 0.004; Figure 4).

### 3.2 Factors associated with PPI prescriptions in patients with ACS and AF

The population was categorized into two groups based on the administration of PPIs, with 254 (47.8%) patients having received PPIs. Univariate analysis revealed several factors significantly associated with PPI use, including NSTEMI, STEMI, paroxysmal AF, the presence of GI disorders, and lower systolic blood pressure. Additionally, patients who underwent PCI/CABG or fibrinolytic, catheter ablation, or who received OAC were more likely to receive PPIs (Table 1).

**Table 1:** Patient clinical characteristics according to PPI use.

Characteristics	Total (*n* = 531)	PPI use (*n* = 254)	No PPI use (*n* = 277)	*p* value
Age, years	73 (64,79)	73 (64,79)	72 (64,78)	0.828
Female	205 (38.6)	96 (37.8)	109 (39.4)	0.713
Types of ACS				
UA	275 (51.8)	97 (38.2)	178 (64.3)	<0.001
NSTEMI	124 (23.4)	70 (27.6)	54 (19.5)	0.028
STEMI	131 (24.7)	86 (33.9)	45 (16.2)	<0.001
Unclassified	1 (0.2)	1 (0.4)	0 (0.0)	0.460
Types of AF				
Paroxysmal	305 (57.4)	165 (65.0)	140 (50.5)	<0.001
Persistent	81 (15.3)	42 (16.5)	39 (14.1)	0.432
Permanent	3 (0.6)	2 (0.8)	1 (0.4)	0.940
Unclassified	142 (26.7)	45 (17.7)	97 (35.0)	<0.001
Comorbidities				
Hypertension	423 (79.7)	196 (77.2)	227 (81.9)	0.171
Diabetes mellitus	240 (45.2)	104 (40.9)	136 (49.1)	0.059
Hyperlipidemia	423 (79.7)	211 (83.1)	212 (76.5)	0.062
Stroke/TIA/systemic embolism	172 (32.4)	81 (31.9)	91 (32.9)	0.831
NYHA/Killip III-IV class	93 (17.5)	45 (17.7)	48 (17.3)	0.906
Malignancy	42 (7.9)	26 (10.2)	16 (5.8)	0.057
Hyperuricemia	78 (14.7)	38 (15.0)	40 (14.4)	0.866
GI disorders	173 (32.6)	151 (59.4)	22 (7.9)	<0.001
Infectious diseases	142 (26.7)	75 (29.5)	67 (24.2)	0.165
Chronic renal dysfunction (eGFR < 60 mL/min/1.73 m^2^)	170 (32.0)	76 (29.9)	94 (33.9)	0.322
SBP at admission, mmHg	130 (117,140)	125 (111,140)	130 (120,140)	0.008
Hemoglobin count at admission, g/L	131.76 ± 20.774	130.61 ± 21.338	132.82 ± 20.224	0.221
Platelet count at admission, ×10^9^/L	193 (154,229)	198 (155.5,235)	190 (152,226.5)	0.231
PCI/CABG/ Fibrinolytic therapy	210 (39.5)	112 (44.1)	98 (35.4)	0.040
Catheter ablation	27 (5.1)	23 (9.1)	4 (1.4)	<0.001
OAC	133 (25.0)	78 (30.7)	55 (19.9)	0.003
NSAID	6 (1.1)	3 (1.2)	3 (1.1)	0.915
ACEI/ARB	331 (62.3)	154 (60.6)	177 (63.9)	0.437
Statins	480 (90.4)	231 (90.9)	249 (89.9)	0.681
β-blockers	379 (71.4)	178 (70.1)	201 (72.6)	0.527
Amiodarone	56 (10.5)	28 (11.0)	28 (10.1)	0.732
Digitalis	28 (5.3)	11 (4.3)	17 (6.1)	0.352
Anti-infective drugs	34 (6.4)	19 (7.5)	15(5.4)	0.332
CHA_2_DS_2_-VASc	5(3,6)	5(3,6)	5(4,6)	0.288
HAS-BLED	2(2,3)	2(2,3)	2(2,3)	0.280

Data are shown as mean ± standard deviation, medians (first to third quartiles) or *n*(%).

ACEI, angiotensin-converting enzyme inhibitor; ACS, acute coronary syndrome; AF, atrial fibrillation; ARB, angiotensin II receptor blocker; CABG, coronary artery bypass graft; eGFR, estimated glomerular filtration rate; GI, gastrointestinal; NYHA, New York heart association; NSAID, non-steroidal anti-inflammatory drug; NSTEMI, non-ST-segment elevation myocardial infarction; OAC, oral anticoagulant; PCI, percutaneous coronary intervention; PPI, proton pump inhibitor; SBP, systolic blood pressure; STEMI, ST-segment elevation myocardial infarction; TIA, transient ischemic attack; UA, unstable angina.

Based on the results of the multivariate logistic regression analysis, several factors were identified as independently associated with the use of PPIs. These factors include UNSTEMI with an adjusted odds ration (OR) of 3.358 [95% confidence interval (CI): 1.819–6.196, *p* < 0.001], STEMI with an adjusted OR of 4.092 (95% CI: 2.177–7.694, *p* < 0.001), paroxysmal AF with an adjusted OR of 1.732 (95% CI: 1.022–2.936, *p* = 0.041), GI disorders with an adjusted OR of 17.625 (95% CI: 9.783–31.751, *p* < 0.001), patients who underwent catheter ablation with an adjusted OR of 13.368 (95% CI: 3.836–46.586, *p* < 0.001) and those who received OAC with an adjusted OR of 1.918 (95% CI: 1.006–3.658, *p* = 0.048). In contrast, systolic blood pressure at admission, as well as the interventions of PCI or CABG or thrombolysis, did not achieve statistical significance in the multivariate model (Table 2).

**Table 2:** Multivariable logistic regression analysis of factors associated with PPI use.

Variable	Adjusted OR (95% CI)	*P*-value
UA	-	-
NSTEMI	3.358 (1.189–6.196)	<0.001
STEMI	4.092 (2.177–7.694)	<0.001
Paroxysmal AF	1.732 (1.022–2.936)	0.041
GI disorders	17.625 (9.783–31.751)	<0.001
SBP at admission	0.990 (0.978–1.002)	0.098
PCI/CABG/Fibrinolytic therapy	1.533 (0.903–2.601)	0.113
Catheter ablation	13.368 (3.836–46.586)	<0.001
OAC	1.918 (1.006–3.658)	0.048

AF, atrial fibrillation; CABG, coronary artery bypass graft; GI, gastrointestinal; NSTEMI, non-ST-segment elevation myocardial infarction; OAC, oral anticoagulant; PCI, percutaneous coronary intervention; PPI, proton pump inhibitor; SBP, systolic blood pressure; STEMI, ST-segment elevation myocardial infarction; UA, unstable angina. Categorical predictors were entered as indicator variables with UA as the reference for ACS (comparisons: NSTEMI vs. UA and STEMI vs. UA) and unclassified as the reference for AF (comparison: paroxysmal vs. unclassified).

## 4 Discussion

To the best of our knowledge, the current study has been the first survey in China to specifically investigate PPI administration among individuals diagnosed with ACS and AF. The main results of this study were as follows: (1) PPIs were prescribed to 47.8% of the patients with ACS and AF at discharge. (2) The administration of PPIs has increased from 2010 to 2018. Patients receiving NOACs were more likely to be prescribed PPIs compared to those taking warfarin. (3) PPI users exhibited a higher prevalence of NSTEMI/STEMI, paroxysmal AF, GI disorders, and were more likely to undergo catheter ablation than non-PPI users. Furthermore, patients receiving OAC therapy were more likely to be prescribed PPIs.

Previous studies have revealed varying incidences of PPI among patients receiving DAPT. A nationwide registry in Danish revealed that only 35% of patients who received DAPT after MI and were at high risk of GIB received PPI therapy based on the guideline criteria, and anticoagulation therapy was excluded (26). A China registry from July 1, 2017 to December 31, 2018 revealed that 63.9% of patients with ACS who received DAPT received PPI within 24 h of admission (27). Additionally, studies analyzing the use of PPIs in patients with AF indicated that 36.9% of elderly Japanese patients with nonvalvular AF were treated with PPIs (28). However, the results of our study vary from those presented in the previous studies. This difference could be attributed to variances in the demographics of the studied populations, changes in guidelines over time, disparities between national protocols, and varying prescribing habits. Although no other study has specifically analyzed PPI administration among patients with ACS and AF, one study investigating the antithrombotic regimen for ACS and AF indicated that 56.4% of patients received PPIs, excluding those who were not indicate for anticoagulation, those with contraindications for antithrombotic regimen, or those with a life expectancy of no more than 6 months (29). These results closely mirrored our findings.

Clinicians are more likely to prescribe PPIs to patients at high risk for GIB. Previous studies have identified older age (>65 years), previous GI disease, and steroid corticosteroids or nonsteroidal anti-inflammatory drug administration, combined with other antithrombotic drugs, as risk factors for GI injury and bleeding among those receiving antithrombotic agents (2, 30, 31). No significant difference in age was found between the two groups, which may be associated with the relatively older age of our study participants. The lack of a significant difference in NSAID usage between the two groups may be due to the limited number of NSAIDs administered to the study participants.

Although anticoagulants play a crucial role in preventing stroke risk among patients with AF, their use significantly increases the risk of GIB. The increased risk of GIB associated warfarin use seems to be connected to its systemic anticoagulant effects through vitamin K-dependent clotting factor inhibition. DOACs may induce GI damage through direct local anticoagulation. Given that dabigatran and factor Xa inhibitors are not fully absorbed after oral intake, the unabsorbed dabigatran etexilate (the prodrug of dabigatran) can become activated within the lumen as it passes through the GI tract (32, 33). Warfarin has been the mainstay of oral anticoagulant therapy for decades. However, the past decade has seen the emergence of DOACs, which present several advantages over warfarin. In 2019, the American College of Cardiology made a class I recommendation favoring the use of DOACs over warfarin for patients with nonvalvular atrial fibrillation. The emergence of NOACs has not only altered the anticoagulant prescribing patterns but may also have impacted the prescribing patterns of PPIs. However, there has been little research on PPI use in these patients.

The current study revealed that approximately 25.0% of the patients received anticoagulation therapy, which was slightly lower than that reported in another study (33.6%) (29). Given that our research primarily focused on investigating PPI administration, patients without indications for anticoagulation therapy or those with contraindications were not excluded. This could potentially explain the lower rates of OAC prescription in our research. The proportion of patients using anticoagulation gradually increased from 2010 to 2018, accompanied by a corresponding increase in PPI usage. Our findings revealed that approximately 32.6% of the patients had GIB. To facilitate the analysis, patients were categorized based on their anticoagulation status. Our results indicated no significant difference in the incidence of GIB between the two groups. However, PPI administration was markedly higher among those receiving anticoagulation therapy.

Our results showed that patients prescribed NOACs were more likely to receive PPIs to prevent GI damage than were those taking warfarin. This result is consistent with those reported in a real-world study on patients with AF in Japan (28) and could be possibly explained by the various GIB risks associated with different OACs. Research indicates that dabigatran and rivaroxaban pose a higher GIB risk relative to warfarin, whereas apixaban was associated with a decrease in GIB risk compared to warfarin (31, 34).

The CHA_2_DS_2_-VAS_C_ scoring system has been ubiquitously used for the evaluation of stroke risk in clinical settings, whereas the HAS-BLED scoring system has been utilized to determine bleeding risk among patients with AF. Our study revealed no correlation between the CHA_2_DS_2_-VAS_C_ or HAS-BLED scores and PPI prescription. One reason for this lack of correlation may be the relatively low actual anticoagulation rate despite the high proportion of patients at high risk in our study. Some studies have indicated that amiodarone, statins, and antibiotics could potentially elevate the risk of bleeding when taken with OACs (35–41). However, the limited usage of OAC in the current study may have caused physicians to overlook the potential bleeding risk caused by drug interactions. Consequently, the use of these medications did not correlate with increased PPI administration.

Antithrombotic regimens for patients with AF and ACS pose a significant challenge in real-world clinical practice and have been a focal point of research in recent years (42–45). The risk of bleeding mainly affects antithrombotic strategies (29). Although the use of PPIs reduces GI damage caused by antithrombotic drugs, the potential risks associated with long-term or excessive PPI use must be considered (46). Current guidelines recommend that PPIs be administered in conjunction with antiplatelet therapy, oral anticoagulants, or both to mitigate the risk of bleeding among patients at high risk for GIB (47). However, the duration of PPI therapy is individualized. We believe it essential to screen patients at high risk of GIB and conduct a dynamic assessment to correct reversible bleeding risk factors and determine the appropriate duration of PPI therapy. Furthermore, enhancing follow-up and monitoring after initiating therapy is crucial. Unfortunately, the administration of PPIs in this specific patient population has not received sufficient attention. Hence, the risks and benefits of combining antithrombotic drugs and PPIs on an individual basis need to be carefully evaluated.

The strength of this research lies in our presentation of the actual clinical use of PPIs among patients with AF and ACS. Our study not only present the current situation of PPI usage but also analyzes the potential factors that influence its utilization. This study provides valuable insights for guiding future interventions aimed at reducing inappropriate PPI administration. However, recognizing the limitations of this study is important. First, this is a single-center study conducted at a tertiary care hospital in Beijing, China. Consequently, the results may not be generalizable to other settings, institutions, or countries. Secondly, it was a retrospective study, and part of patients were excluded because of incomplete information or death, which may lead to selection bias. Furthermore, although this study examined the current status of PPI use in patients diagnosed with ACS and AF, it did not assess the appropriateness of PPI prescriptions, which should be a focus of future research. Additionally, the lack of follow-up data restricts the generalizability of the conclusions. Moreover, previous studies have indicated that gastroesophageal reflux disease can impact coronary artery lesions (48). Exploring these interactions would be an interesting future research direction.

## 5 Conclusions

The results of this study indicate that approximately 50% of patients with AF and ACS were prescribed a PPI at discharge. The administration of PPIs has increased from 2010 to 2018. Patients receiving NOACs were more likely to be prescribed PPIs compared to those taking warfarin. PPI users exhibited a higher prevalence of NSTEMI/STEMI, paroxysmal AF, GI disorders, and were more likely to undergo catheter ablation. Additionally, patients receiving OAC therapy were more likely to be prescribed PPIs. We advise conducting a dynamic assessment to correct reversible bleeding risk factors and to determine the appropriate duration of PPI therapy for patients with ACS and AF. We hope to promote the rational use of PPIs to prevent GIB while minimizing adverse reactions.

## Data Availability

The datasets presented in this study can be found in online repositories. The names of the repository/repositories and accession number(s) can be found in the article/Supplementary Material.
